# Dynamic energy correlation analysis of *E. coli* aspartokinase III and alteration of allosteric regulation by manipulating energy transduction pathways

**DOI:** 10.1002/elsc.202000065

**Published:** 2021-03-02

**Authors:** Shizhen Wang, Chengwei Ma, An‐Ping Zeng

**Affiliations:** ^1^ Department of Chemical and Biochemical Engineering College of Chemistry and Chemical Engineering Xiamen University Xiamen P. R. China; ^2^ Institute of Bioprocess and Biosystems Engineering Hamburg University of Technology Hamburg Germany

**Keywords:** allosteric regulation, aspartokinase III, dynamic energy correlation, energy redistribution, energy transduction pathway

## Abstract

Conformational change associated with allosteric regulation in a protein is ultimately driven by energy transformation. However, little is known about the latter process. In this work, we combined steered molecular dynamics simulations and sequence conservation analysis to investigate the conformational changes and energy transformation in the allosteric enzyme aspartokinase III (AK III) from *Escherichia coli*. Correlation analysis of energy change at residue level indicated significant transformation between electrostatic energy and dihedral angle energy during the allosteric regulation. Key amino acid residues located in the corresponding energy transduction pathways were identified by dynamic energy correlation analysis. To verify their functions, residues with a high energy correlation in the pathways were altered and their effects on allosteric regulation of AKIII were determined. This study sheds new insights into energy transformation during allosteric regulation of AK III and proposes a strategy to identify key residues that are involved in intramolecular energy transduction and thus in driving the allosteric process.

**Abbreviations**
AKIII: *E. coli* aspartokinase III
PScN: protein side‐chain networks
RMSD: root mean‐square deviations.
SMD: steered molecular dynamics

## 1 INTRODUCTION

As a typical dynamical model for investigating the relationship between protein structure and function, allosteric proteins have been studied for decades and are still of great interest [1, 2, 3]. Although static structures are known for many proteins, the functions of proteins are determined ultimately by their dynamical characteristics and energy landscape [4]. In this context, allosteric proteins are of particular interest because conformational changes are the basis of allosteric regulation which is dynamic and involves both signal and energy transductions. So far, several methods have been developed to study signal and energy communication during allosteric regulation of proteins [5, 6]. For example, energy exchange network of inter‐residue interactions based on CURrent calculation for proteins (CURP) was proposed for the analysis of a neuronal protein PSD‐95 [7]. An energy dissipation model has been proposed to investigate *Escherichia coli* (*E. coli*) aspartokinase III (AK III) mutations for better understanding the desensitization of product feedback inhibition via allostery [8].

As a key enzyme in amino acid biosynthesis, aspartokinase catalyzes the phosphorylation of the amino acid aspartate. This reaction is the first step in the biosynthesis of three essential amino acids: methionine, lysine, and threonine. In *E. coli*, there are three independently regulated isozymes of aspartokinase, each of which is specific to one of the three downstream biochemical pathways [9]. This allows independent regulations of synthesis of methionine, lysine, and threonine respectively. Among them, the enzyme AKIII coded by the gene *lysC* is l‐lysine‐sensitive. It consists of a dimer of protein encoded by *lysC*. In the presence of lysine, it can adopt an inactive tetrameric conformation [10].

For better understanding the mechanisms of allosteric regulation, *E. coli* AKIII is employed as a model system in this study. We aimed to reveal the structural and energetic features of the key residues in the dynamic process of allosteric regulation of this enzyme. To this end, steered molecular dynamics simulations were performed to figure out residues involved in the allosteric regulation and this was followed by energy transduction analysis. In addition, sequence conservation analysis of the aspartokinase family was carried out to calculate the conservation score of each amino acid residue and to identify conserved sites. Key amino acid residues that participate in the allostery process were identified to be those holding a high energy correlation score with both of the lysine binding site and the aspartic acid binding site. To verify the predictions, AK III mutants with mutation of key residues were experimentally studied.

**PRACTICAL APPLICATION**

In this work, we combined steered molecular dynamics simulations and sequence conservation analysis to investigate the conformational changes and energy transformation in the allosteric enzyme aspartokinase III (AK III) from *Escherichia coli*. Energy correlation analysis indicates dynamic transformation and compensation between electrostatic energy and dihedral angle energy during allosteric regulation of AKIII. Key residues involved in the allostery are identified to exhibit a high energy correlation score with both of the lysine and aspartic acid binding sites. Energy transduction between the binding sites is triggered by ligand binding/leaving, leading to amplitude structural motions in the protein. The energy transduction pathways were redesigned to modulate the allosteric regulation and validations were conducted by site‐directed mutagenesis. This work highlights the importance of structural dynamics and energy landscape of conformations and provides new insights for kinase engineering and design.

## 2 MATERIALS AND METHODS

### 2.1 System preparation

The x‐ray diffraction structures of *E. coli* AK III were retrieved from the Protein Data Bank (PDB). In this work, the crystal structure of the T‐state (PDB code 2J0X) was employed for study. After deleting the lysine located at the regulatory site, the T‐state structure was neutralized by adding sodium and chlorine ions with an ionic concentration of 0.5 mol/L and solvated in a rectangular box of TIP3P water molecules with a minimum solute‐wall distance of 20 Å. The solvated system was energy‐minimized by 5000 steps employing the software of NAMD.

### 2.2 Molecular dynamics simulations

In this work, steered molecular dynamics (SMD) simulations were performed by auto‐searching the pathway of energy minimization. With a periodic boundary condition in the NPT ensemble, Langevin dynamics applied at 310 K with the damping coefficient of 5.0 ps‐1 and constant pressure of 1 atm. No constraint was applied to the protein during the molecular dynamics simulations. A time step of 2 fs was used and the coordinates of the simulated complexes were saved every 1.0 ps. The simulations lasted for 10 ns and were performed employing the software Amber with the CHARMM27 force field. The Collective Variable‐based Calculations (Colvars) module embedded in the NAMD software was used to conduct the SMD.

A harmonic restraint was set with the force constant of 10.0 kcal/mol and the ligand was pulled out of the binding pocket until its center from that of the protein was extended by 10 Å. Parameters were as follow: forceConstant 10.0; centers 9.0310; targetCenters 19.0310; targetNumSteps 5000000. Lysine was completely pulled out from AKIII at the end of simulation. In total, 2000 snapshots were obtained. More details of dynamic simulation are submitted as Supplementary Material.

Protein side‐chain networks (PScN) were then constructed by considering amino acid residues as nodes; edges between the nodes were added on the basis of non‐covalent interactions and side‐chain interaction between them [8]. A residue‐based cut‐off for the non‐covalent interactions was 20 Å.

### 2.3 Dynamic correlation analysis

Dynamic correlation analysis was employed to identify residues that mediate the energy transduction within AKIII. This approach is based on the correlation coefficients between each pair of residues which were obtained from the trajectory of steered molecular dynamics simulations. The correlation coefficients are calculated according to the following equation [8]:

(1) $$C\left(Rs,Rt\right)=\frac{\sum \left(Rs-{\left\langle Rs\right\rangle }_{N}\right)\left(Rt-{\left\langle Rt\right\rangle }_{N}\right)}{\sqrt{{\sum \left(Rs-{\left\langle Rs\right\rangle }_{N}\right)}^{2}}\sqrt{{\sum \left(Rt-{\left\langle Rt\right\rangle }_{N}\right)}^{2}}}$$

where *C*(*R*s*,R*t) is the correlation coefficient between the residues *s* and *t*. *R*s and *R*t are the coordinates of the residues *s* and *t*; 〈*R*s〉*_N_* and 〈*Rt*〉*_N_* are the average coordinates of residues *s* and *t* calculated based on the whole trajectory and *N* is the total number of snapshots recorded in the trajectory. The correlation coefficients of distance, energy and energy change in small time scale between each pair of residues were calculated using Matlab.

Correlation analysis of energy was carried out by using Matlab function corr2 (A, B), which computes the correlation coefficient between arrays A and B. The so‐called C Value, which indicates the energy correlation score of residue X with both residue A and B, was calculated using Equation 2:

(2) $$\begin{array}{ccc} & & C=\\ & & \sqrt{\left|Cor(ResidueA,ResidueX)\right|\times \left|Cor(ResidueX,ResidueB)\right|} \end{array}$$

### 2.4 Conservation analysis

Conservation analysis of AKIII (PDB ID: 2j0x) was carried out by using the ConSurf webserver (http://consurf.tau.ac.il/2016/) with HMMER as the Homolog search algorithm and the HMMER E‐value was set to be 0.0001.There are 902 HMMER hits of sequences. Amino acid sequences which are similar to AK III in the PDB were collected and multiply aligned using CSI‐BLAST and MAFFT, respectively. The evolutionary conservation of each amino acid position in the alignment was calculated using the Rate4Site algorithm. The algorithm takes explicitly into account the phylogenetic relations between the aligned proteins and the stochastic nature of the evolutionary process. Multiple sequence alignment was built using MAFFT. The homologues were collected from SWISS‐PROT. Homolog search algorithm is BLAST. PSI‐BLAST E‐value was 0.001. Maximal %ID between sequences is 95 Minimal %ID for homologs is 35.

### 2.5 Enzyme activity assay and site‐directed mutagenesis

Activity assay and site‐directed mutagenesis of AK III were carried out according to Chen et al. [10]. Specifically, AK III encoded by the gene *lysC* was obtained from *E. coli* K12 MG1655 genome and inserted into pET22b at the *Nde*I and *Xho*I restrict enzyme sites. For over‐expression and purification of the wild‐type AK III and relevant mutants, the wild‐type *lysC* gene was cloned to pET‐22b(+) (Novagen, Darmstadt, Germany) with the introduction of an additional His‐tag at the C‐terminal to generate the plasmid pET22‐lysC. Strain cultivations were performed in shake flasks at 30°C and 250 rpm. The activity was measured with a method adapted from Blank and Wright (1954). The preparation for the enzyme reaction assay was composed of 200 mM Tris‐HCl (pH 7.5), 10 mM MgSO_4_·6H_2_O, 10 mM aspartate, 10 mM ATP, 160 mM NH_2_OH·HCl (neutralized with KOH), and appropriate amounts of enzyme. Total reaction volume was 500 μL. After incubation at 37°C for 30 min, the reaction was stopped by mixing with 1 mL of a 5% (wt/vol) FeCl_3_ solution, and the absorbance at 540 nm was monitored.

Site‐directed point mutations were generated by PCR reaction using QuickChange site‐directed mutagenesis kit (Agilent Technologies, Germany). PCR reactions were carried out with a pair of synthetic complementary oligonucleotides containing the desired mutation as primers and a plasmid with the inserted gene as a template. A mutant plasmid containing staggered nicks was generated by extension of the oligonucleotide primers during temperature cycles. All PCR reactions were performed using Pfu or Phusion polymerase obtained from Fermentas (Germany) with a DNA Thermal Cycler DOPPIO (VWR, Germany).

## 3 RESULTS AND DISCUSSION

### 3.1 Steered molecular dynamics simulations of *E. coli* AK III

**FIGURE 1:**
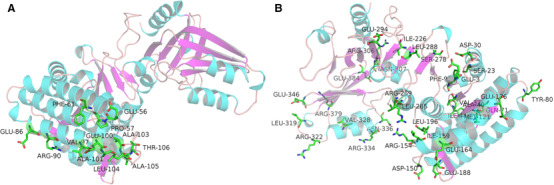
(A) Residues with a RMSD value higher than 10 Å referred from the steered molecular dynamics simulations; (B) Residues with total energy change higher than 8 kJ/mol (ID3, 9, 23, 30, 37, 40, 44, 71, 80, 176, 196, 226, 265, 269, 288, 294, 306, 307, 319, 322, 328, 336, 384, 121, 150, 154, 159, 164, 188, 278, 334, 346, 379)

Steered molecular dynamics simulations apply forces to a molecule in order to manipulate its structure by pulling it along desired degrees of freedom. This approach can be used to study the allosteric regulation in a large timescale [7]. In this study steered molecular dynamics simulation was employed to study the process of pulling lysine out of the ligand binding site of AKIII. Backbone root mean‐square deviations (RMSD) were calculated based on the trajectory with the starting configuration as the reference and all coordinate frames from the trajectories were first superimposed on the reference conformation. Structural analysis based on RMSD was carried out. Residues with a RMSD value higher than 10 Å include 54, 55, 59, 84, 88, 95, 98, 99, 101, 102, 103, and 104 (Figure 1A).

Energy variation analysis was also carried out for each residue. Residues with a total energy change of more than 8 kJ/mol are shown in Figure 1B and they are supposed to trigger energy redistribution for driving the allosteric regulation of AKIII. It is noteworthy that most of the residues did not exhibit a high RMSD value, indicating that the structure/position‐based parameters such as RMSD are not able to explore the energy response mechanism that undergoes an allosteric process.

### 3.2 Energy transformation during the allosteric process

For a given protein, the energy function that defines its so‐called energy landscape is a combination of the conformational energy and non‐bond energy [13]. The conformational energy is related to the covalent interactions, namely, the energy of bonds, bond angles and dihedral angle energy. The non‐bond energy includes the van der Waals', and electrostatic energy. In the case of AKIII, the correlation coefficient of conformational energy and non‐bond energy of all residues was calculated to be –0.45 during the steered dynamic process. Correlation coefficient which is less than –0.3, indicates that transformation and compensation occur among these two energy items during the allostery procedure.

Subsequently, correlation analysis was carried out to define the major driving energy. Correlation analysis of all the energy items listed in Table 1. From Table 1, it can be seen that the correlation coefficient between non‐bond energy and electrostatic energy is as high as 0.999, which indicated that the electrostatic energy is the main source for non‐bond energy redistribution [14]. Then, the correlation coefficient of conformational energy and dihedral angle energy was calculated to be 0.627, indicating that dihedral angle energy makes great contributions to the conformational energy change. And the correlation coefficient of electrostatic energy and dihedral angle energy is –0.414, implying that the electrostatic interaction energy and dihedral angle energy are transformed to each other and may drive the allosteric modulation. A recent study also proposed that allostery in the well‐studied PDZ domain is driven by changes in electrostatic effects rather than solely changes in dynamics [15].

**TABLE 1:** Correlation coefficients among different items of energy obtained from correlation analysis of energy transformation and redistribution during steered molecule dynamic simulation

Energy items	Bond energy	Angles energy	Dihedral angle energy	Conformational energy	Electrostatic energy	Van der Waals' energy	Non‐bond energy
Bond energy	1	0.2997	0.2145	0.5884	−0.3797	0.0993	−0.3760
Angles energy	0.2997	1	0.1943	0.8354	−0.2518	0.1370	−0.2462
Dihedral angle energy	0.2145	0.1943	1	0.6268	−0.4139	0.1535	−0.4079
Conformational energy	0.5884	0.8354	0.6268	1	−0.4592	0.1868	−0.4518
Electrostatic energy	−0.3797	−0.2518	−0.4139	−0.4592	1	−0.0513	0.9991
Van der Waals' energy	0.0993	0.1370	0.1535	0.1868	−0.0513	1	−0.0084
Non‐bond energy	−0.3760	−0.2462	−0.4079	−0.4518	0.9991	−0.0084	1

**FIGURE 2:**
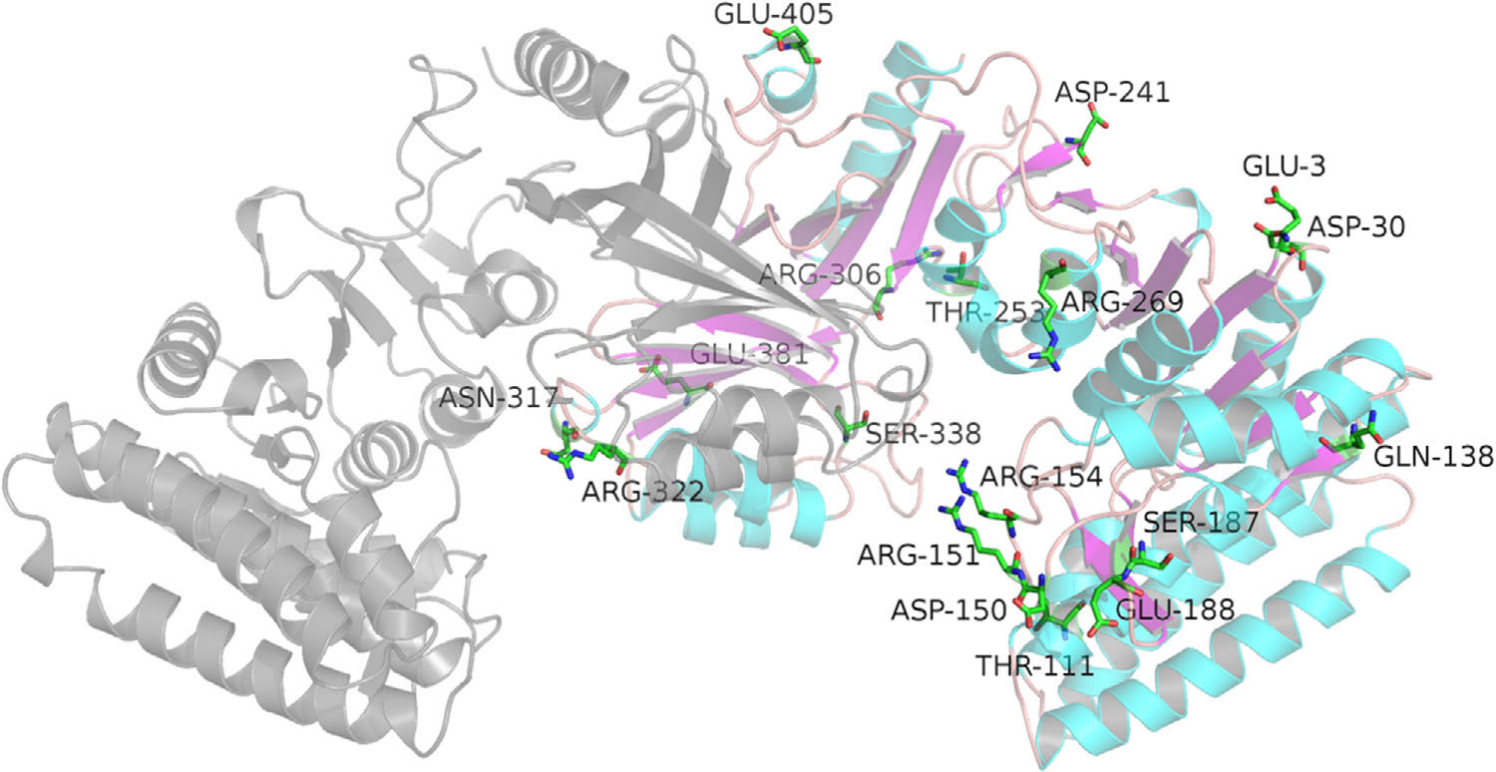
Residues with electrostatic energy change more than 5 kJ/mol

Due to the compensating effects of different energy items, namely electrostatic interaction energy and dihedral angle energy, the total energy of the protein is perturbed to a much lower extent. Electrostatic interaction energy becomes the key determinant in distinguishing the structural ensembles between the ligand bound and unbound states, and in elucidating the allosteric modulation. The electrostatic interaction redistribution is a common feature among “dynamic‐driven” allosteric proteins [16]. However, electrostatic interactions are the main source for non‐bound energetic redistribution, while van de Waals interactions and H‐bonded networks are both involved in energetic redistribution to some extent [17]. Residues with an electrostatic energy change of more than 5 kJ/mol are showed in Figure 2 (residue ID 3, 322, 30, 111, 138, 150, 151, 154, 187, 188, 241, 253, 269, 306, 317, 338, 381, 405). Among them, mutation of 253 from Thr to positively charged Arg was reported to be able to deregulate the allostery of AK III [18]. These results indicated that the mutation can change the energy transformation and then affect the allostery.

### 3.3 Energy change and conservation of the binding sites

The energy change of residues in the starting and final states of the steered MD calculations was calculated. The changes of lysine, aspartate and ADP binding sites during the steered molecule dynamic simulation are listed in Tables 2, 3, 4. For the lysine binding site, there are significant changes of the electrostatic energy and dihedral angle energy of residue D340, which were –4.656 and 5.204 kJ/mol, respectively. Residue 338 experienced a large electrostatic energy change of –5.134 kJ/mol. According to the crystal structure of AKIII, D340 and E346 can form two salt bridges with lysine. Mutation of S338L, E346R and V339A were reported to deregulate allosteric process by affecting the binding of lysine [10]. Obviously, redistribution of internal energies happened within the protein, especially the electrostatic energy.

**TABLE 2:** Energy change of lysine binding residues (kJ/mol)

Residue ID	Bond energy	Angles energy	Dihedral angle energy	Electrostatic energy	Van der Waals' energy	Total energy
318	3.276	2.585	0.897	−1.240	1.320	6.838
321	3.698	−1.098	−2.461	−2.170	0.329	−1.702
324	0.765	3.844	−3.360	−0.758	0.056	0.547
325	−0.617	0.508	2.133	−0.642	−1.490	−0.108
338	2.259	0.356	−0.667	−5.134	2.755	−0.432
339	1.937	2.045	−4.372	1.603	0.290	1.502
340	1.683	3.485	5.204	−4.656	−1.265	4.452
345	−0.385	1.588	1.410	1.120	0.023	3.755
346	4.522	1.331	0.735	0.781	1.889	9.258

**TABLE 3:** Energy change of residues in the aspartate binding site (kJ/mol)

Residue ID	Bond energy	Angle energy	Dihedral angle energy	Electrostatic energy	Van der Waals' energy	Total energy
8	0.413	9.457	−0.467	−1.612	−1.047	6.744
45	2.641	−0.504	2.294	3.697	−0.149	7.979
198	3.496	−3.054	−0.028	2.959	−2.274	1.099
199	−0.512	3.177	0.743	0.055	1.857	5.321
201	−0.934	3.150	−1.119	−4.695	−0.027	−3.625
202	−1.995	2.895	4.351	−0.517	−0.256	4.479
222	0.261	1.852	2.227	3.277	−0.490	7.127
39	0.701	1.684	−0.433	2.523	1.232	5.707
119	1.014	−0.205	0.450	−4.943	1.340	−2.344
184	0.312	0.471	2.273	−0.513	−1.084	1.459

**TABLE 4:** Energy change of residues in the ADP binding site (kJ/mol)

Residue ID	Bond energy	Angle energy	Dihedral angle energy	Electrostatic energy	Van der Waals' energy	Total energy
227	−0.552	−1.894	−2.998	2.152	−0.638	−3.930
232	1.605	−0.597	−3.068	−1.342	−2.302	−5.704
257	−1.516	3.465	2.746	1.075	0.331	6.100
258	3.013	−3.922	0.986	−0.637	−0.265	−0.825
221	−3.959	2.281	−1.835	2.776	1.216	0.478
202	−1.995	2.895	4.351	−0.517	−0.256	4.479

For the aspartate binding site, there are significant electrostatic energy changes in residues 30, 199 and 201, which were –8.241, –4.943, –4.695 kJ/mol, respectively. S201 binds with both aspartate and ADP. However, there is much less electrostatic energy change in the ADP binding site, except the residue 201, which is also shared with the aspartate binding site. Therefore, the aspartate binding site undergoes a greater energy change than the ADP binding site.

As for the conservation of the residues located at the binding sites, the lysine binding site is less conserved than the aspartate/ADP binding sites according to their average conservation scores. For the lysine binding site, its average conservation score was –0.849, while for the binding sites of aspartate and ADP, they were –1.062, and –0.949, respectively. Several studies have been reported about altering the inhibitor from lysine to norlecuine et al. by mutation of the ligand binding site [10, 11]. Engineered proteins were catalytically active and delivered new functional allostery with switched ligand specificity [12].

### 3.4 Electrostatic energy correlation analysis

The release of lysine from the binding pocket causes energy redistribution, which is associated with the conformational change. As a converse process, this is also true for the binding process of the ligand into the pocket. To figure out how this perturbation is propagated within the protein, correlation coefficients of the electrostatic energy were calculated for each residue. Then, all amino acid residues were clustered using the software Cytoscape by considering amino acid residues as nodes and electrostatic energy correlation scores as edges.

**FIGURE 3:**
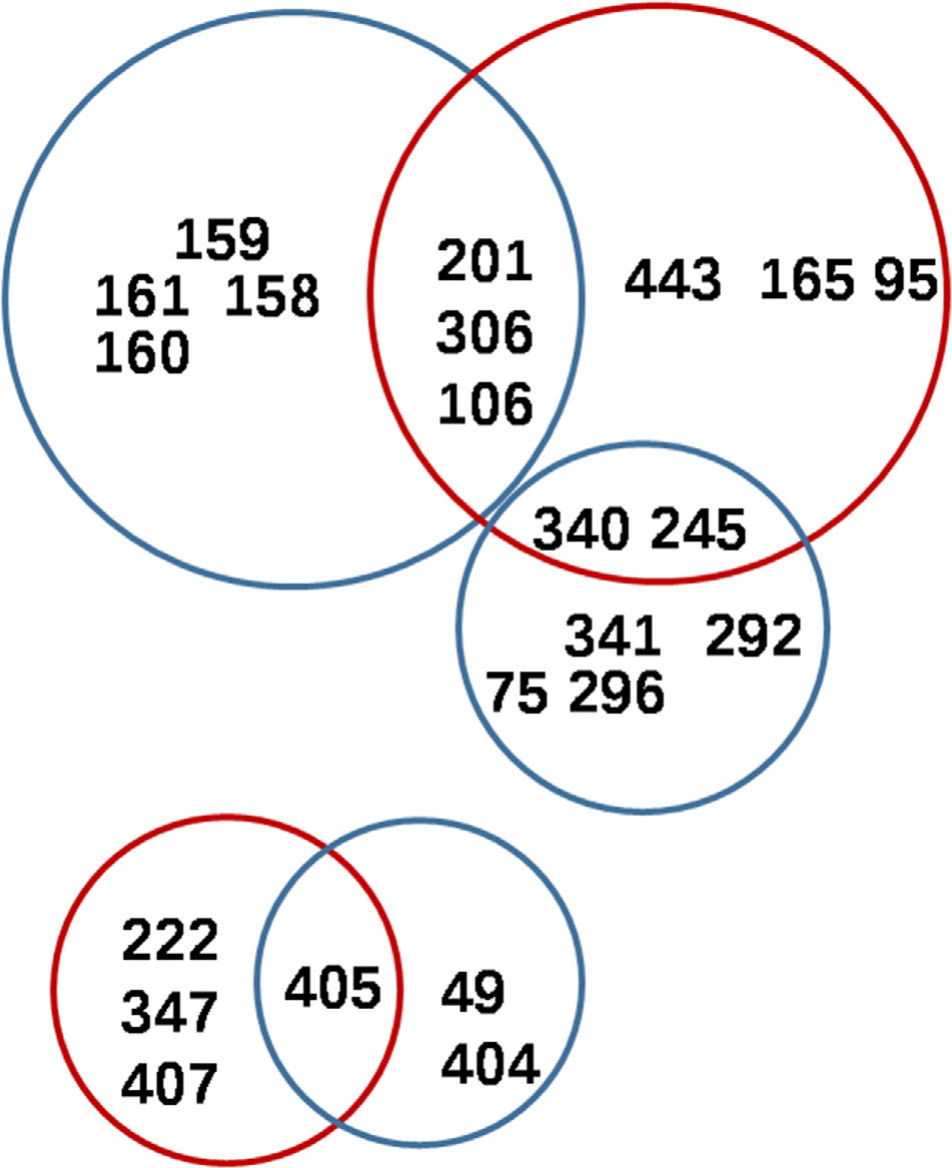
Key residues referred from intersects of correlation coefficient of electrostatic energy less than –0.5 (blue circles) and more than 0.5 (red circles)

Residues 341, 340, 292, 75, 245 and 296 were clustered with electrostatic energy correlation less than –0.5, which obviously indicated an electrostatic energy transduction among them. And residues 443, 245, 340, 165, 306, 106, 201, 95 were clustered with an electrostatic energy correlation coefficient which is more than 0.5, which indicated that they may act as an energy pool for driving the conformational change. Of those residues, F407 was reported to be in the protein dynamical modules based on energy dissipation [8]. V347M has previously been reported to be able to deregulate AKIII through the approach of random mutation [17]. As given in Figure 3, residues 106, 201, 306, 245, and 340 have high electrostatic energy correlation scores. Among them, residue 201 is located in the aspartate binding site. These results indicated that correlation analysis of electrostatic energy can provide a highly sensitive probe toward identifying the key residues in the energy redistribution [15].

### 3.5 Energy redistribution pathways

To reveal how the energy is transferred in proteins through the interaction network, 12 pathways were proposed from the key residues of lysine binding site (Residue A: S338, D340, E346) to aspartate binding site (Residue B: S39, G119, D202, S201) were studied based on energy correlation coefficients of each residues. Only residues that obtain high energy correlation coefficients with both of the beginning residue and the end residue will obtain high C scores. Residues with a C score of higher than 0.3 are considered as key residues in the energy transduction pathway.

Residues in the pathway between E346 to S201 showed the highest correlation among all the 12 pathways revealed by SMD. This indicates that it is the main pathway of energy transfer. The pathway from E346 to S39 includes 26 residues with C score more than 0.3. The pathways from D340 to S39 and S201 also showed a high correlation. Due to the fact that S39 and S201 are the binding residues shared by both of the aspartate and the ADP binding sites, it can be assumed that energy redistribution pathways which connect the lysine and aspartate binding sites . And the 345‐222 route is also a key signal transfer route according to the previous work done by Ma et al. [8].

**FIGURE 4:**
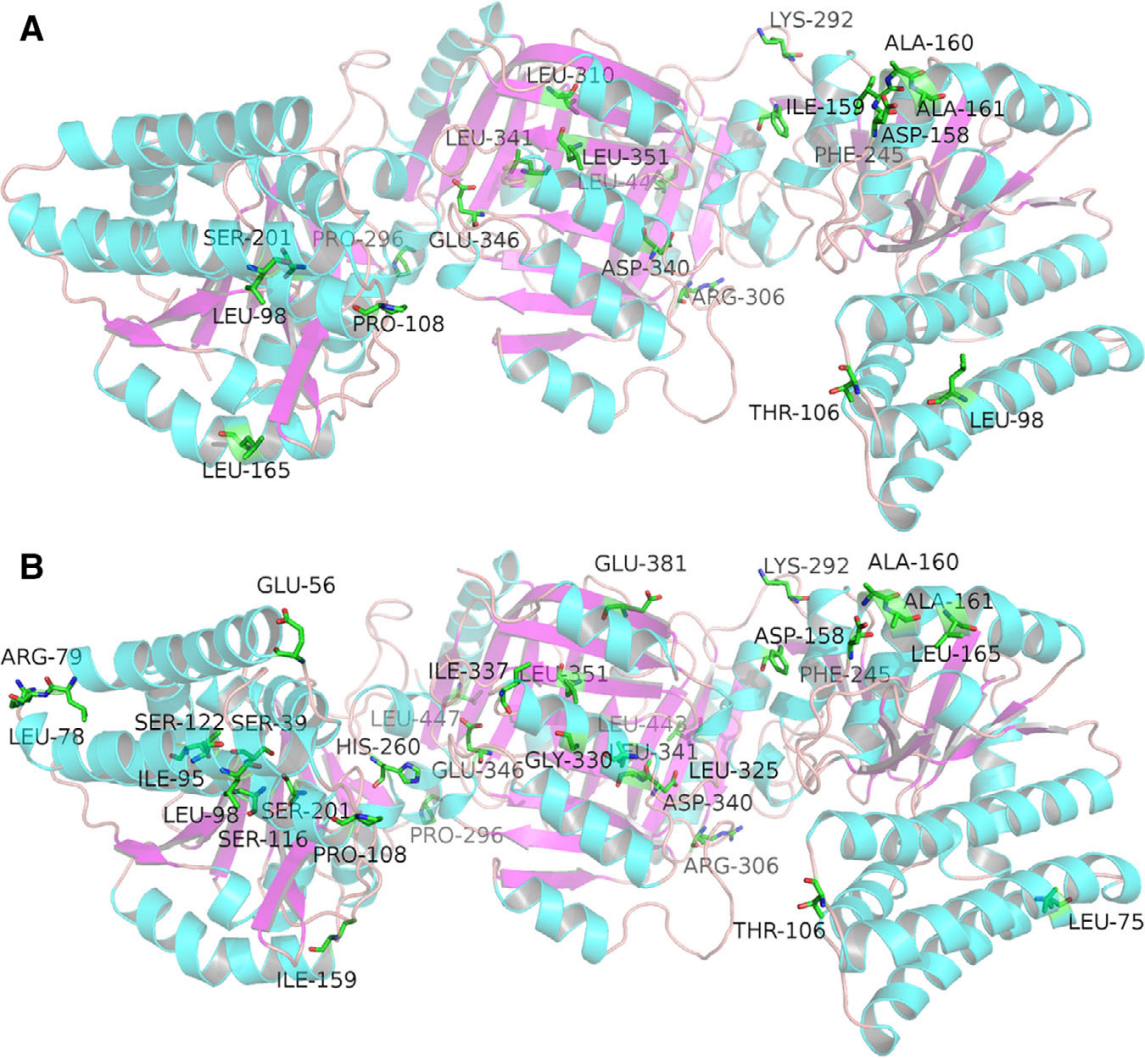
(A) Key residues in the energy transfer route of 346‐201 with high correlation coefficients of electrostatic energy more than 0.3; (B) Key residues in the energy transfer route of 340‐201 with high correlation coefficients of electrostatic energy more than 0.3

Shared amino acid residues in the routes of D340 to S201, E346 to S201 and E346 to S39 includes (ID 106, 158, 160, 165, 245, 260, 292, 296, 306, 341 and 443), which formed two big clusters. Key residues which shared in all of the four pathways (D340, E346 to S201, S39) were residues 106, 165, 245, 292, 296, 306 and 443. The average conserved scores of the residues (ID 106, 165, 245, 292, 296, 306 and 443) which shared in many pathways have a high value of –0.0471. This is moderately conserved, while those residues which only exist in a single pathway exhibited less conserved. For example, 19 residues in the pathway of E346 to S201 with an average value of 0.316 (Figure 4). This indicates that the shared residues in the energy transduction pathway are relatively more conserved.

Besides, the residues 106, 245 and 306 also show a high value of correlation coefficient for the electrostatic energy which is more than 0.5. Residue 306, which belongs to the ACT1 domain, contributes the major energy transduction in the pathway based on the electrostatic energy redistribution. These results are in agreement with the dynamical mechanism for allosteric transition and inhibition by lysine in AKIII proposed by Kotaka et al. [9].

### 3.6 Experimental verification by site‐directed mutagenesis

**FIGURE 5:**
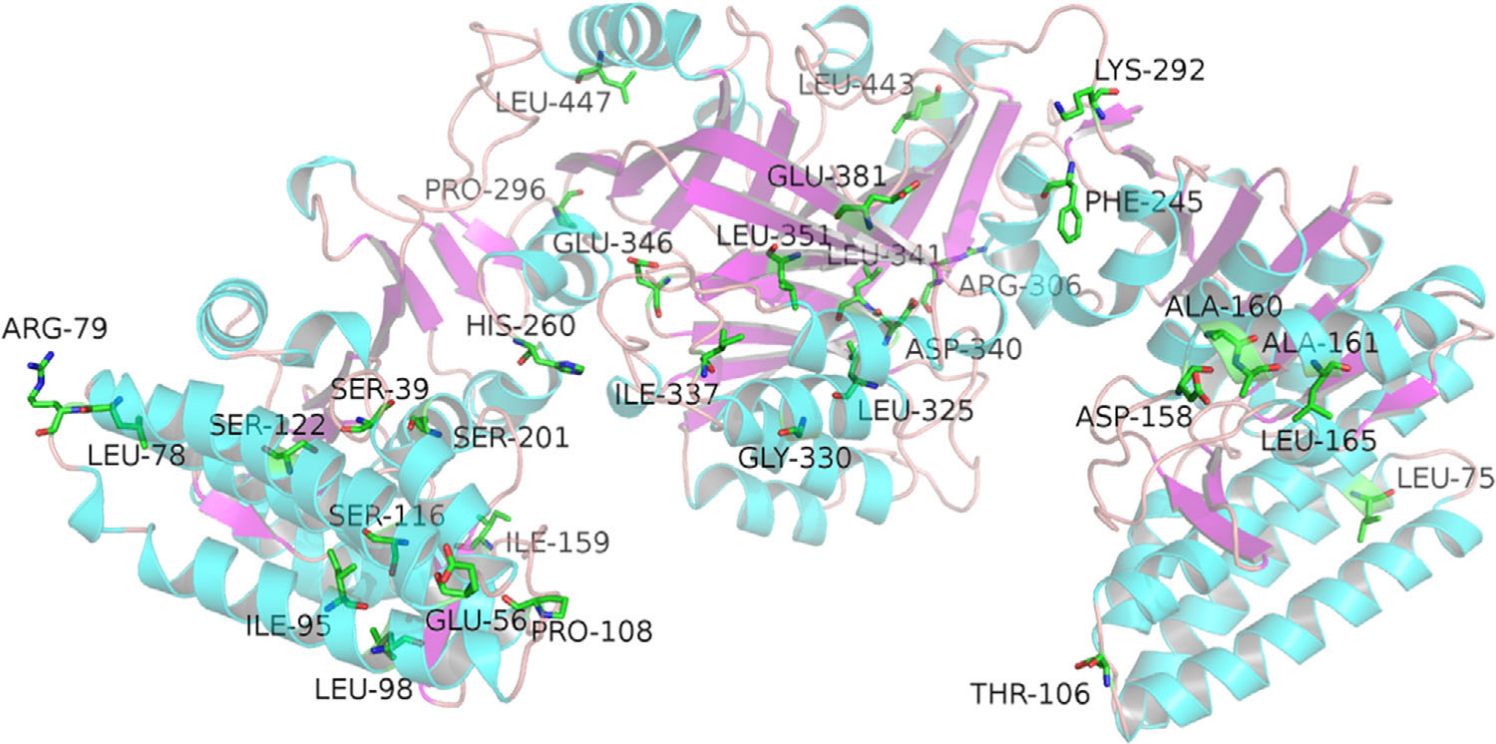
Residues selected for mutation in this work (Residues 106, 165, 292, and 296)

Most of the mutations reported in the literature (Table 5) which are able to deregulate the feedback inhibition of AK III are around the lysine binding site [19]. Few mutations were reported that are within the energy transduction pathways. In this study, key residues in the energy transduction pathways were selected according to the analysis of energy correlation. Several residues that are not located within the active site motifs were selected for mutation (Figure 5). Thus, the experimental measurements that follow in this work would not arise the problem of changing substrate binding. Allosteric regulation has been affect by point mutations in the energy change pathway.

**TABLE 5:** Reported mutations of AKIII

Mutation site	Effect on inhibition	Reference	Mutation site	Effect on inhibition	Reference
E93W	Enhancement	[9]	F329R	Deregulation	[10]
E100D	Enhancement	[9]	I337P	Deregulation	[18]
E113K	Enhancement	[9]	S338L	Deregulation	[18]
A237V	Enhancement	[9]	V339A	Deregulation	[18]
M251P	Deregulation	[10]	I344P	Deregulation	[10]
T253N	Enhancement	[9]	E346R	Deregulation	[10]
T253R	Deregulation	[18]	R416A	Deregulation	[10]
F275W	Enhancement	[9]	N424A	Deregulation	[10]
G277A	Enhancement	[9]	N426A	Deregulation	[10]
R300C	Deregulation	[18]	I427 P	Deregulation	[10]
R305A	Deregulation	[18]	C428R	Deregulation	[10]
H320A	Deregulation	[18]	E436A	Deregulation	[10]

**TABLE 6:** Relative activities of mutations of AKIII

Mutations	Conserved scores	Relative activities
Wild type	–	100.00
T106G	−0.2880	57.43
L165G	0.8030	23.81
F245G	−0.8590	12.35
K292D	1.1990	48.68
K292D/P296G	1.1560	27.52
R306E	−0.3040	100.91
R379H	0.632	56.89
L443G	−0.7910	8.48

**FIGURE 6:**
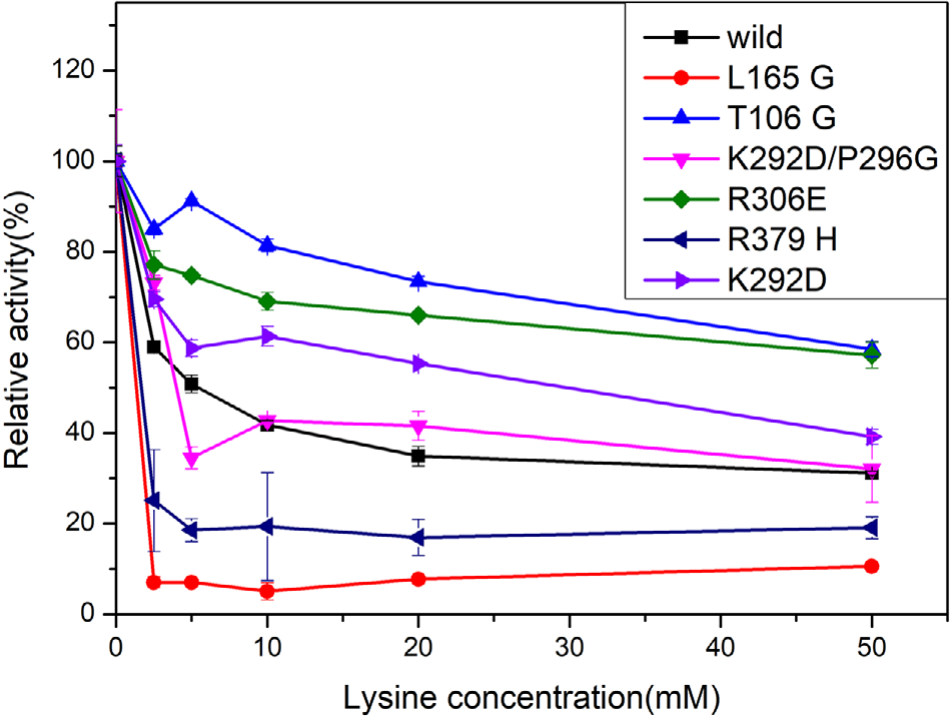
Relative activities of the wild type and mutants of AKIII with lysine as the effector. The relative activities were calculated based on their activity without lysine

Five key residues have been mutated, namely 106, 165, 245, 292, and 296, which were shared in the all four pathways of D340, E346 to S201, and S39. The relative activities were calculated based on their initial activity without lysine. Mutation of K292D deregulated the inhibition effect, while double mutations of K292D/P296G have almost the same inhibition effect as with the wild type. Residue 245 was highly conserved with a conservation score as –0.8590. The mutation of F245G greatly decreased the enzyme activity, indicating that 245 is a key residue for enzyme activity even it is far away from the binding sites. The mutation of 165 from Leu to the less bulk Gly enhanced the inhibition effect by decreasing Dihedral angle energy. The mutation of T106G deregulated the inhibition effect greatly, which verified the energy transduction pathway we predicted. The mutation of K292D changed the positively charged residue into a negative one, indicated interference of electrostatic interaction deregulated inhibitor effect. These results indicated that electrostatic interaction is a key factor for energy transduction of the allosteric system for AKIII (Figure 6).

## 4 CONCLUDING REMARKS

With AKIII from *E. coli* as a model system, the dynamic energy transformation between electrostatic energy and dihedral angle energy during allosteric regulation was explored based on steered molecular dynamics simulations. A strategy for identification of key residues involved in energy transduction during the allosteric process was proposed based on the dynamic energy correlation analysis. Mutations were conducted and their effects on allosteric regulation were determined. The results demonstrated that electrostatic energy is the key driving force for allosteric regulation. The intramolecular energy transduction that drives the allosteric process is crucial for a more holistic and better understanding allosteric mechanism. Energetic features of the key residues in dynamic allosteric regulation process can apply for better understanding of the allosteric regulation of AKIII and for designing and optimizing of allosteric systems with a better performance.

## CONFLICT OF INTEREST

The authors have declared no conflict of interest.

## FUNDING

The authors gratefully acknowledge the support of K. C. Wong Education Foundation and DAAD (no. 91700128). This work was also supported by the National Natural Science Foundation of China (no. 21776233, 22078273), Natural Science Foundation of Fujian Province of China (No. 2018J01013), Fundamental Research Funds for the Central Universities (no. 20720200038, 20720170033).

## DATA AVAILIBILITY STATEMENT

The data that support the findings of this study are available from the corresponding author upon reasonable request.
